# The American Public Health Association

**Published:** 1873-05-15

**Authors:** 


### Society Reports.

### THE AMERICAN PUBLIC HEALTH ASSOCIATION.

The first annual meeting of this Association was held in Cincinnati, commencing Thursday, May 1st, 1873. The meeting was called to order at 9 A. M., the President, Dr. Stephen Smith, of New York, in the chair.

The Secretary, Elisha Harris, M.D., having communicated to the President his inability to be present, Dr. E. H. Jones, of New York, was chosen to fill that office *pro tem.* After announcing the order of business submitted by the Executive Committee, the Secretary read letters from members who were prevented from attending the session. These letters were from President White, of Cornell University, N. Y.; C. R. Agnew, M.D., N. Y.; Prof. Cappell, University of Virginia; J. C. Nutt, M.D., New York (since deceased); F. Peyre Percher, M.D., Charleston, S. C.; Wm. F. Wraag, M.D., Charleston, S. C.

The following members were present at the morning session : Dr. Stephen Smith, of New York, Chairman; Dr. John H. Rauch, Chicago, Ill.; Dr. C. C. Cox, Washington, D. C.; Dr. Edwin M. Snow, Providence, R. I.; Dr. C. B. White, New Orleans, La.; Dr. Wm. Clendenin, Cincinnati, Ohio; Dr. J. E. Reeves, Wheeling, W. Va.; Dr. Moreau Morris, New York; Dr. John M. Woodworth, Washington, D. C.; Dr. E. H. Janes, New York; Dr. Thos. M. Logan, Secretary of Board of Health, Sacramento, California; Dr. Edward Jarvis, Dorchester, Mass.; Dr. John M. Toner, Washington, D. C.; Dr. A. N. Belle, Commissioner of Quarantine, Brooklyn, N. Y.

The President delivered a valuable and interesting address on the "Limitations and Modifying Conditions of Human Longevity as the Basis of the Practical Application of Sanitary Knowledge." After an interesting survey of the whole field of human existence, embracing climate, food, habits, and disease in various forms, inherited and contracted, and showing the modifying influences of enlightened knowledge on the part of the individual, and of communities. The speaker summed up with an extended notice of the more important methods for applying sanitary knowledge with the best effect.

Dr. Toner, of Washington, was next requested to read an abstract of the Statistics of the Boards of Health of the United States. These statistics had been gathered together through the co-operation and assistance of the Bureau of Education at Washington. The following are the principal results derived from the figures : Total No. of Health Boards reporting, 118. No. of Health Boards reporting from the State of Alabama, 2; California, 2; Connecticut 3; Delaware, 1; District of Columbia, 1; Florida, 1; Georgia, 2; Illinois, 10; Indiana, 8; Iowa, 3; Kansas, 5; Kentucky, 1; Louisiana, 1; Maine, 4; Maryland, 1; Massachusetts, 11; Michigan, 8; Minnesota, 1; Mississippi, 1; Missouri, 4; Nebraska, 1; New Hampshire 2; New Jersey, 5; New York, 11; Ohio, 8; Pennsylvania, 5; Rhode Island, 1; Sonth Carolina, 1; Texas, 1; Vermont, 1; Virginia 6; West Virginia, 2; Wisconsin, 4. Total, 118.

Total No. of said Boards reported, 690; No. of Boards not reporting members, 7; No. of Boards exercising authority over cities, only 90; having extra urban authority, 22; State Boards, 3; not respecting extent of jurisdiction, 3; reporting population within bounds of jurisdiction, 116; total population reported, 7,356,560; date of oldest Board organized, A.D. 1780.

No. Boards organized before 1800, 2; between 1800 and 1810, 2; 1810 and 1820, 2; 1820 and 1830, 2; 1830 and 1840, 6; 1840 and 1850, 7; 1850 and 1860, 23; 1860 and 1870, 30; since 1870, 27; not reporting date of organization, 17.

*Method of Selecting Board.*—Appointed by medical societies, 1; by city authorities, 96; by election, 13; by President of United States, 1; by Governors, 3. No. of Boards not paid, 62; Secretaries only salaried, 6; Health officers salaried, 9; other officials salaried, 6; in which two or more are salaried, 30.

The composition of the Boards is as follows: Having only medical members, 16; a majority of medical members, 14; a minority of medical members, 50; no medical members, 14.

No. reporting register of births kept, 17; marriages, 10; deaths, 33. No. required to report dates, ages, and causes of death, 33.

*Water Supply, Drainage, Paving, etc., of Cities.*—No. Boards reporting water works, 72; water supply abundant and pure, 89; underground drainage, 76; paved streets, 76.

*Highest and lowest points above sea level.*—No. cities having highest point less than 100 feet above, 11; 100 to 500, 34; 500 to 1000, 24; 1000 or more, 3. No. cities having lowest point less than 10 feet above, 19; 10 to 100, 7; 100 to 500, 8; 500 to 1000, 23; 1000 or more, 3.

The Secretary was then called upon to read a report on a Uniform System of Registration of Causes of Death throughout the United States, submitted by Chas. P. Russell, M.D., of New York.

After a preliminary consideration of the obvious necessity for the establishment of some more complete and uniform system of registration of the causes of mortality throughout the United States, the author proceeded to a detailed consideration of the system of nosology devised and carried out with so much success by Dr. Farr. This classification has received the endorsement of several international statistical congresses; and Dr. Russell thinks undoubtedly possesses, in its practical bearings upon public health, a vast superiority over any other system ever put into operation. It exhibits clearly, and at a glance, the relative prevalence of the same classes of diseases in the greatest imaginable variety of circumstances.

In the first and principal group of this classification are comprised the most formidable diseases with which sanitary science has to grapple, and the investigation of which, with reference to their prevention, modification, or annihilation, has, within a few years, become the subject perhaps of the most momentous importance to the human race, both in a physical and moral sense. These are the epidemic, endemic, parasitic, inoculated, and epizootic disorders, as well as those resulting from the deprivation of sustenance and abuse of drink; all being classed under the general designation *Zymotic*. It is eminently proper that affections of this character should be grouped together, notwithstanding peculiarities which seem to separate them as clearly from one another as from those of different classes. The resemblance in their origin, course, influence, and effect, is of vastly more consequence than the diversity in their individual traits.

Besides the Zymotic disorders, there is, as Dr. Farr expresses it, a legion of diseases never halting, and not so much controlled by external circumstances as sporadic diseases, or ordinary maladies of every day occurrence. These prevail universally; are sometimes due to hereditary taint,' sometimes apparently spontaneous, or traceable to a variety of exciting causes more or less insidious; but they are usually regarded as capable of direct propagation. The chief, and most generally fatal, of these are the constitutional disorders, including the tuberculated, scrofulous, and the diathetic.

Next come the great class of Local disorders—affection of special organs—appropriately grouped in distinct systems. In another order are classed the Developmental diseases, or those arising from abnormal action of the formative, reproductive, or nutritive processes.

It is to be presumed, the author says, that objections to certain details of the foregoing classification will occur to some persons; but we believe that in its main features it cannot but prove acceptable to all. We do not maintain that it is absolutely perfect; but its defects become insignificant when we contemplate it as accomplishing its great purpose of throwing light upon the mysterious laws of national disease. This system has long been in vogue in Great Britain, and has been employed in the metropolis of your own country for seven years.

If our accumulations of facts are to have any value, it must be not only on account of their intrinsic truth, but also from their deductions when compared with those of other countries. In this manner, statistics of mortality assume vast importance, and present for our consideration manifold questions of a physical and social character. But putting aside the broad and philosophic view of the importance of mortality statistics, it is obvious that the application of their deductions must be of immense benefit to the physician, merely as a practitioner. This was proved even as long ago as the time of Sydenham, who inculcated the doctrine that the treatment of diseases should have reference not only to the immediate symptoms and to the season, but also to the morbific constitution of the year and the locality. It has been remarked by a distinguished author that "man is not born, does not live, does not suffer, does not die in the same manner on all parts of the earth. Births, life, disease and death, all change with the climate and soil, all are modified by race and nationality."

In conformity, therefore, with these principles enunciated, we urge that your Association should recommend to all organizations or persons having supervision of death returns in cities, towns, or villages of the United States, the adoption, at as early a date as may be expedient (say June 1st, 1874), of forms, classified, of causes of death, with slight modifications.

In conclusion, the author suggested some additions and modifications.

A consideration of this subject involved and called up at once a discussion of the much vexed question of the nomenclature or classification of diseases.

The chair said the paper was deeply interesting, and urged a full and free discussion on the subject, as there was no doubt of the great necessity for a full and intelligent registration. He was of opinion that the existing system was practically useless.

Dr. J. J. Woodward, of the United States army, regretted that the author of the paper was not present. He felt the necessity for a registration that would be uniform, and therefore apply as well to America as to England. He was under the impression that the nomenclature of the College of Physicians has been adopted by the Registrar General of England, and he was of opinion that it was excellent. He had adopted it because he felt it his duty to do so. There was still another classification in the field, and it was of that he desired most to speak, namely, an American report of classification made at the American Medical Association, which he regarded as not as good as the first one spoken of. There were differences between it and the English, but they were not such as commanded respect. The speaker gave instances of a purely professional and scientific character, showing these differences. What was wanted greatly was a classification of diseases that would bring epidemics into one class. He would rather stick to the old alphabetical arrangement of the names of diseases than to adopt the Philadelphia monstrosity, and he hoped this Association would not adjourn until it adopted some expression of opinion on the subject.

Further discussion of the subject was postponed until the afternoon session, and the Association adjourned until 2½ o'clock, P.M.

AFTERNOON SESSION.

The Association was called to order again at 2½ o'clock, P.M., when the President announced the following names of physicians recommended to the Executive Committee for election to membership: Drs. John A. Murphy and R. D. Mussey, of Cincinnati; Drs. Baker, L. P. Jaynes, and F. H. Davis.

The report of the Committee was adopted, and the gentlemen named were declared members of the Association.

The reading of the papers was then continued, C. C. Cox, M. D., of Washington, D. C., presenting one on the subject of "The Necessity of a National Sanitary Bureau." It was a comprehensive document, following the history of national sanitary movements from the first measures adopted by the Roman Empire to the present time. It also contained many suggestions as to the means of securing the health of the nation, noting the origin and effects of disease in all classes of life, laying particular stress upon ventilation, quarantine, drainage, and sewerage. The scope of the Sanitary Bureau should include mortuary and meteorological statistics.

After some little discussion following the reading of the paper, the following resolution, offered by Dr. Bell, was adopted:

*Resolved*, That, in the judgment of this Association, the establishment of a "National Sanitary Bureau," with relations to the general Government similar to those of the Bureau of Agriculture and Education, is highly desirable as a means of promoting sanitary science and the protection of public health.

Dr. Woodward resumed his remarks of the forenoon on classification and nomenclature, and offered the following:

*Resolved*, That in our opinion it is of the utmost importance that a common nomenclature and classification be adopted for the purposes of the registration of deaths and diseases, and especially by all English speaking people.

*Resolved*, That we regard the nomenclature and classification of diseases proposed by the College of Physicians of London, which has been extensively adopted in Great Britain and the United States, as more likely to be generally adopted for these purposes than any other system yet proposed, and that we hereby recommend its provisional adoption in the United States.

Dr. Snow thought that much could not be gained by differing from from the English classification.

Dr. Jarvis suggested the difficulties arising in the way of a perfect classification, inasmuch as people will not get sick according to science. He thought the idea of including all diseases classified as impossible.

On motion, the first resolution of Dr. Woodward was carried.

After some discussion, the second resolution was also adopted.

Dr. Cox offered the following, which was adopted:

*Resolved*, That Dr. J. J. Woodward, Dr. E. Jarvis and Dr. E. M. Snow be appointed a committee to communicate the foregoing resolutions to Dr. Simpson, of London, and to negotiate for the representation of this Association in the first decennial revision of the nomenclature.

Dr. Toner offered the following, which was adopted:

*Resolved*, That the American Public Health Association deems it opportune that an international medical congress be requested to assemble during the American Centennial of 1876, in Philadelphia, to consider and adopt a nomenclature and uniform classification of diseases in registration of deaths throughout the world.

Dr. Rauch offered the following, which was adopted:

*Resolved*, That Boards of Health be requested to make out their reports on vital statistics for the period beginning in January and ending in December, and not for any financial and municipal year.

The next paper presented was from Dr. Jarvis, mainly made up of statistics showing the effects which public calamities, such as war, famine, etc., have in decreasing the marriage and birth rates.

Dr. Snow offered the following, which was adopted:

*Resolved*, That the Committee on Registration be requested to prepare and present to this Association a blank for uniform weekly and monthly reports of mortality in our cities.

Adjourned to meet at 8 o'clock P.M.

EVENING SESSION.

The Association re-assembled at 8 o'clock, and heard an interesting paper read by the Secretary *pro tem.*, Dr. E. H. Janes, of New York, on the horse epizootic, in which the disease was traced throughout its course, commencing in Toronto. The Chairman stated that the facts were derived in pursuance of the directions of the New York Board of Health, and by it transmitted to the Association for its information and use. The course of the epizootic was marked out on a map hung up for inspection during the lecture, and its effects were exhibited by drawings from *post mortem* examinations.

Adjourned to meet at 9 o'clock A.M.

---

SECOND DAY'S PROCEEDINGS.

The order of reports was resumed at 10 o'clock, in the reading of that prepared by Dr. Elisha Harris, of New York, on "Areas of Sanitary Administration, and the Practical Application of General Laws and Local Ordinances Relating to the Public Health." This paper was read by the President, Dr. Smith, and stated that health laws were proved by experience to be practically useful, acceptable, and well administered, in proportion as the inhabitants of communities are intelligently concerned about the causes for which such laws and regulations have been called into existence. In the United States this truth is practically more important, and has fewer exceptions than in most other countries. All sanitary regulations exercised by Government authorities in confronting the march of cholera proved unavailing, except when communities applied them intelligently. The only real security against the march of disease lies in the intelligent support of the local and general health laws by the local and State authorities. This train of argument was continued in advocating Central Boards of Health.

The second paper of the morning was that prepared and read by Wm. Clendenin, M. D., of Cincinnati, on "The General Causes of Disease."

Drs. Cox, Bell, Jarvis and White spoke in highly commendatory terms of Dr. Clendenin's paper; and Dr. Toner expressed the hope that something would be done by the Association to give it practical effect. The Association then adjourned until 2½ o'clock, to avail themselves of an invitation from the officers of the Cincinnati Hospital to visit that institution. An invitation from the House of Refuge authorities was accepted with thanks.

At the conclusion of the report, Dr. Woodward offered the following resolution, which was adopted:

*Resolved*, That a committee consisting of Drs. Elisha Harris, Dorman B. Eaton, John Ordronaux, and Stephen Smith, be appointed to consider the best plan for the appointment and organization of National, State and local Boards of Health, and that the discussion of this report be made a special order of business.

---

THIRD DAY'S PROCEEDINGS.

The Association assembled again at 9 o'clock.

After the transaction of some miscellaneous business, Dr. Toner presented the following resolution:

WHEREAS, It is important that Boards of Health of cities should at all times have as definite information as to the number, condition, and distribution of their population as practicable; and whereas it has been found practicable in other countries to collect such information, approximating correctness, through the aid of circulars properly prepared and distributed, which mode is attended with little if any cost, and can be performed by the ordinary form of the various Boards of Health; therefore be it

*Resolved*, That local Boards of Health be recommended, whenever practicable, to take a census of their population annually, on the 1st of January, by the instantaneous mode—that is, through the aid of circulars, with blank inquiries upon all points of special interest to vital statistics and sanitary conditions, these blanks to be previously distributed to each family and housekeeper within the city, to be filled up by them at a fixed hour on the same day—the same to be called for on the following day by officers of the Board of Health, or by the police of the city, or by any other method that may be desirable.

After some discussion on minor points, the above was unanimously adopted.

The regular proceedings of the Association were dispensed with at this point, on motion of Dr. Toner, in order that the members and visitors present might hear an address from Professor J. S. Newberry, of Cleveland, on the "Homes of the American People."

Dr. Jarvis followed with some general remarks, closing in offering a resolution as follows:

*Resolved*, That the thanks of this Association are hereby tendered to Prof. J. S. Newberry, for his able and exceedingly instructive address on the topography, geology, and climatic conditions of the unoccupied Territories of the United States, with reference to their adaptation to the health of the people hereafter to occupy them.

Dr. Toner seconded the above, and further considered the subject of our future population in a brief speech.

The resolution was then adopted unanimously.

ADJOURNMENT.

On motion, it was resolved that when this Association adjourns, it adjourn to meet on the second Wednesday of September next, at Providence, R. I.

Resolutions of thanks were also passed to Dr. Murphy and to the Cincinnati Hospital for handsome receptions; to Dr. Clendenin for his personal attentions; and to the Cincinnati College and Cincinnati Law School for the use of their halls.

The Association then adjourned, to meet as above determined.

